# Human and mouse non‐targeted metabolomics identify 1,5‐anhydroglucitol as SGLT2‐dependent glycemic marker

**DOI:** 10.1002/ctm2.470

**Published:** 2021-06-27

**Authors:** Ben A. Kappel, Julia Moellmann, Kirsten Thiele, Matthias Rau, Anna Artati, Jerzy Adamski, Bart Ghesquiere, Katharina Schuett, Francesco Romeo, Robert Stoehr, Nikolaus Marx, Massimo Federici, Michael Lehrke

**Affiliations:** ^1^ Department of Internal Medicine 1, University Hospital Aachen RWTH Aachen University Aachen Germany; ^2^ Metabolomics and Proteomics Core, Helmholtz Zentrum München German Research Center for Environmental Health (GmbH) Neuherberg Germany; ^3^ Institute of Experimental Genetics, Helmholtz Zentrum München German Research Center for Environmental Health Neuherberg Germany; ^4^ Institute of Biochemistry, Faculty of Medicine University of Ljubljana Ljubljana Slovenia; ^5^ Department of Biochemistry, Yong Loo Lin School of Medicine National University of Singapore Singapore; ^6^ VIB Metabolomics Expertise Center VIB Center for Cancer Biology Leuven Belgium; ^7^ KU Leuven Metabolomics Expertise Center, Department of Oncology Katholieke Universiteit Leuven Leuven Belgium; ^8^ Department of Systems Medicine University of Rome Tor Vergata Rome Italy; ^9^ Center for Atherosclerosis Policlinico Tor Vergata Rome Italy

**Keywords:** biomarker, metabolomics, SGLT2, type 2 diabetes

**Abbreviations**
1,5‐AG: 1,5‐anhydroglucitol
AUC: area under the curve
BCAA: branched‐chain amino acid
EMPA: empagliflozin treatment
HbA1c: glycated hemoglobin
MI: myocardial infarction
ROC: receiver operating characteristic
SGLT: sodium‐glucose cotransporter

Dear Editor,

Metabolomics provide a powerful tool in the search for biomarkers and pathways related to a disease or in response to a drug therapy. Metabolites are appealing biomarkers in metabolic diseases, because a defective, disease‐related pathway may lead to accumulation or deficiency of certain metabolites. In this study, we combined three different non‐targeted serum metabolomics datasets in human and mice to explore pathways and biomarkers related to diabetes. In a comparative analysis between mice and men, we discovered metabolites related to a diabetic phenotype. We next assessed those biomarkers in another cohort of patients with type 2 diabetes (T2D). In mice and humans including a placebo‐controlled trial, we report the impact of sodium glucose cotransporter (SGLT) 2 inhibitor empagliflozin on previously identified glycemic marker 1,5‐anhydroglucitol (1,5‐AG).

Full methods are described in detail in the Supporting Information. Db/Db mice and Db/+ control mice were fed a high‐fat diet ± empagliflozin.^1^ Two prospective cohorts for non‐targeted metabolomics were recruited: (1) 37 patients with and without T2D hospitalized for myocardial infarction (MI) (Table S1, Figure S1) and (2) 25 patients with T2D included in an empagliflozin registry (Table S2, Figure S2).^2^ A targeted analysis of 1,5‐AG was performed in a placebo‑controlled, randomized, double blind human trial with empagliflozin (n = 42).^3^

We first explored serum metabolomics data of the MI cohort and Db/Db versus Db/+ mice (Figure 1A,B; Table S3 and Table S4). Considering the top 10 most significant metabolites by *P*‐value, both data sets showed a clear separation of diabetes versus non‐diabetes (Figure 1C,D; Tables S3 and S4). Metabolites associated to branched‐chain amino acid (BCAA), glutathione, acylcarnitine metabolism, and 3‐hydroxy fatty acid metabolism were altered in diabetes versus non‐diabetes (Tables S3 and S4). In both data sets, our non‐targeted approach found monosaccharide 1,5‐AG to be reduced by the diabetic phenotype (Figure 1C,D).

**FIGURE 1:**
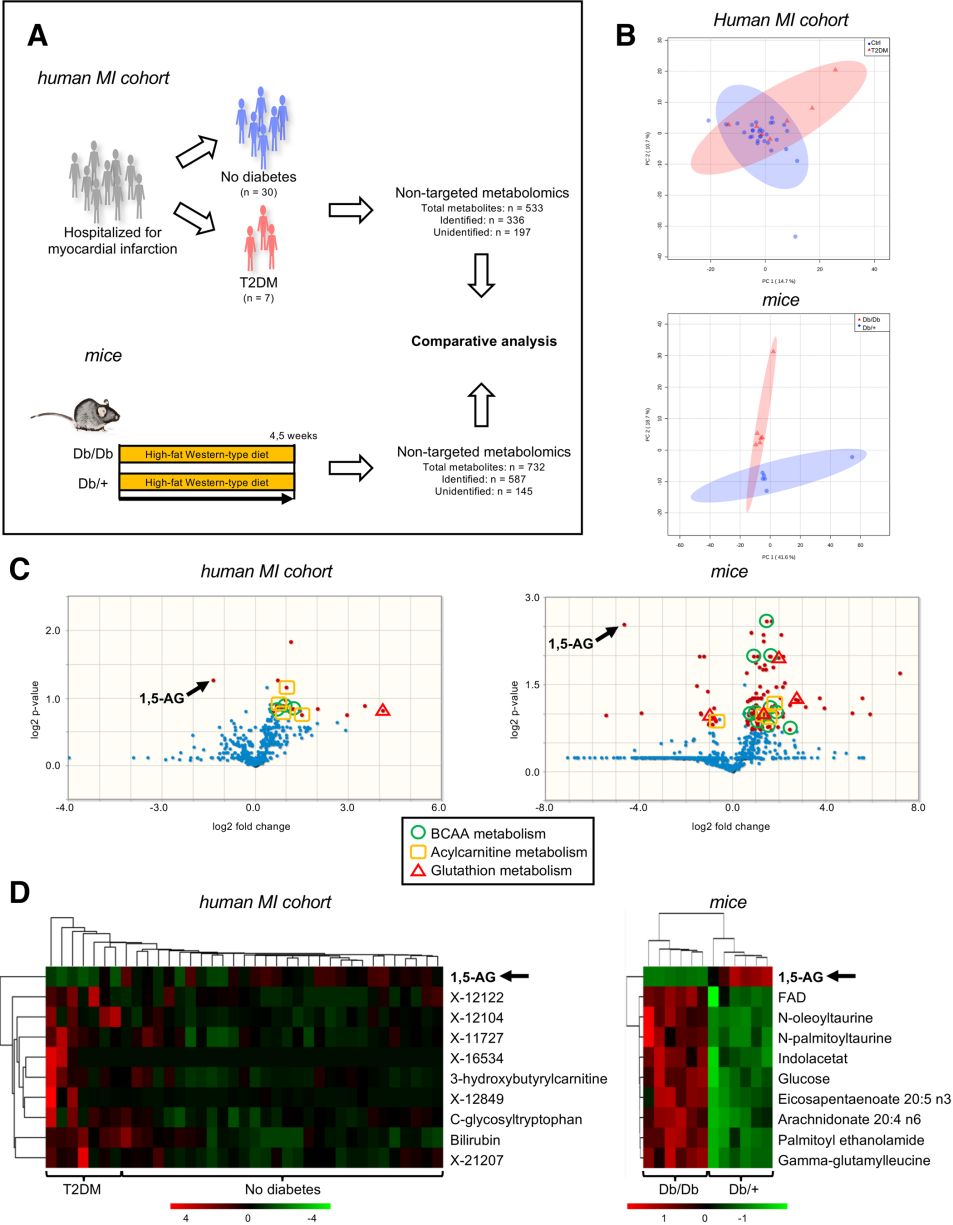
Serum metabolomics reveal biomarkers for diabetes in human and mice. (A) Comparative analysis of non‐targeted serum metabolomics from human and mice ± diabetes. Human myocardial infarction (MI) cohort: Patients hospitalized for MI (n = 37). Mice: diabetic (Db/Db) versus non‐diabetic mice (+/Db)(n = 6 per group). T2D: Type 2 diabetes mellitus. (B) Principal component analyses of metabolomics from human (upper) and mice (lower). (C) Volcano plots of human MI cohort serum metabolomics (left) and mice serum metabolomics (right). Red dots indicate metabolites with fold‐changes over 1.5 and significant P‐values by two‐sided t‐test corrected for multiple testing by false discovery rate (FDR < 0.3). 1,5‐AG: 1,5‐anhydroglucitol. BCAA: branched‐chain amino acid. (D) Top 10 metabolites by two‐sided t‐test corrected for multiple testing by false discovery rate (FDR <  0.3) of human MI cohort serum metabolomics (left) and mice serum metabolomics (right). X‐“ are unidentified metabolites

Consistent with 1,5‐AG being reduced in the diabetic stratum in mice and men (Figure 2A), we found a strong negative correlation between 1,5‐AG and serum glucose (Figure 2B). ROC analysis and multivariate regression analysis identified 1.5‐AG as strong, independent biomarker for diabetes (Figure 2C; Table S5).

**FIGURE 2:**
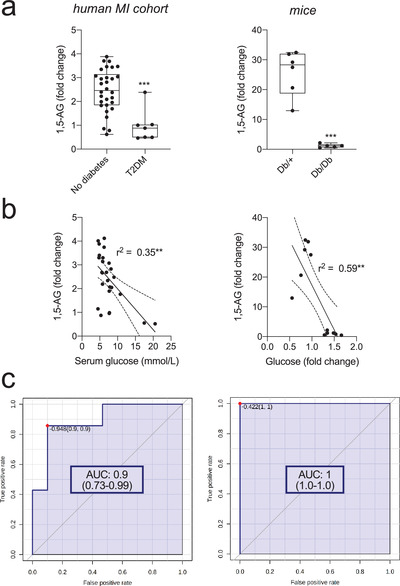
1,5‐Anhydroglucitol is a biomarker for diabetes in human and mice. (A) 1,5‐Anhydroglucitol (1,5‐AG) is reduced in patients and mice with diabetes. ***P < 0.001 by two‐sided *t*‐test. (B) 1,5‐AG correlates with serum glucose in human (right) and mice (left). Pearson correlation: *r* ^2^. **P < 0.01. (C) ROC curves to evaluate 1,5‐AG as biomarker for diabetes (human (right) and mice (left)). AUC with 95% confidence interval

To confirm these results, we investigated another metabolomics dataset of the empagliflozin registry. Patients were grouped in tertiles on basis of their baseline glycemic control in respect to glycated hemoglobin (HbA1c) (Figure 3A; Table S2). According to our previous findings, we identified 3‐hydroxy fatty acids as well as acylcarnitines to be positively correlated with glycemic markers (Figure 3B). 1,5‐AG was identified as the only metabolite among the top 25 metabolites with negative correlation to HbA1c and glucose (Figure 3B; Figure S3, Tables S6 and S7). Using multivariate regression analysis the correlation between HbA1c and 1,5‐AG remained significant (Table S8). We have previously reported the impact of empagliflozin treatment on the serum metabolomics profile.^2^ Unexpectedly, although patients showed better glycemic control under empagliflozin treatment, levels of 1,5‐AG were largely diminished (Figure 3C,D). At baseline, before empagliflozin treatment, 1,5‐AG did well differentiate between good glycemic control (HbA1c < 60 mmol/mol) and poor glycemic control (> 60 mmol/mol; Figure 3E). However, this differentiation by 1,5‐AG was lost during empagliflozin treatment attributable to very low levels of 1,5‐AG observed in all three groups – which proved independency of HbA1c (Figure 3E). Consequently 1,5‐AG only correlated with HbA1c and serum glucose at baseline but not under conditions of empagliflozin (Figure 3F,G). We next asked whether a similar decline of 1,5‐AG levels does also occur in response to SGLT2 inhibition in diabetic mice (Figure 3H). We found already low 1,5‐AG levels in the diabetic environment to be further reduced in response to SGLT2 inhibition (Figure 3I,J).

**FIGURE 3:**
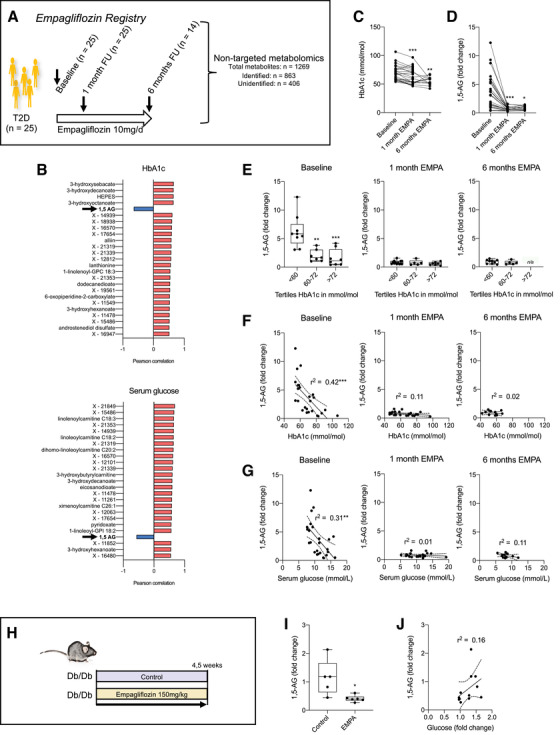
SGLT2 inhibitor empagliflozin confounds glycemic marker 1,5‐anhydroglucitol in diabetic patients and mice. (A) Serum metabolomics performed in the Empagliflozin Registry. Patients with type 2 diabetes mellitus (T2D) at baseline before empagliflozin treatment (n = 25) and at follow‐up (FU) 1 month (n = 25) and 6 months (n = 14) after initiation of empagliflozin 10 mg/day. (B) Changes in HbA1c after treatment with empagliflozin. **P < 0.01, ***P < 0.001 versus baseline by mixed‐effects analysis with Tukey's post hoc test. EMPA: empagliflozin treatment. (C) Changes in 1,5‐anhydroglucitol (1,5‐AG) after treatment with empagliflozin. *P < 0.05, ***P < 0.001 versus baseline by mixed‐effects analysis with Tukey's post hoc test. (D) Top 25 metabolites correlating with glycemic markers in the Empagliflozin Registry (Pearson correlation with *P* < 0.05 and FDR < 0.3). Upper graph: HbA1c. Lower graph: Serum glucose. “X‐“ are unidentified metabolites. (E) Levels of 1,5‐AG as fold changes among different tertiles of HbA1c (in mmol/mol) at baseline (left), 1 month (middle) and 6 months (right) of empagliflozin treatment (EMPA). **P < 0.01, ***P < 0.001 versus baseline by 1‐way ANOVA with Tukey's post hoc test. n/a: no data available. (F) Pearson correlation analysis of 1,5‐AG and HbA1c before (left) and after empagliflozin treatment (middle and right). ***P < 0.001. (G) Pearson correlation analysis of 1,5‐AG and serum glucose before (left) and after empagliflozin treatment (middle and right). **P < 0.01. (H) Diabetic mice (Db/Db) fed a high‐fat Western diet ± empagliflozin 150 mg/kg (n = 6 per group). (I) Levels of 1,5‐anhydroglucitol (1,5‐AG) as fold changes between Db/Db mice ± EMPA. *P < 0.05 by two‐sided *t*‐test. (J) Pearson correlation analysis of 1,5‐AG and serum glucose in Db/Db mice ± EMPA

In a placebo‑controlled, randomized, double blind human trial (Figure 4A),^3^ 1,5‐AG was significantly reduced by empagliflozin treatment , but not by placebo (Figure 4B). While at baseline (before empagliflozin), 1,5‐AG showed a strong correlation to HbA1c and serum glucose, these correlations lost under conditions of empagliflozin treatment (Figure 4C,D). These results confirm SGLT2 inhibition to reduce 1,5‐AG levels, possibly by increasing its urinary excretion in mice and men.

**FIGURE 4:**
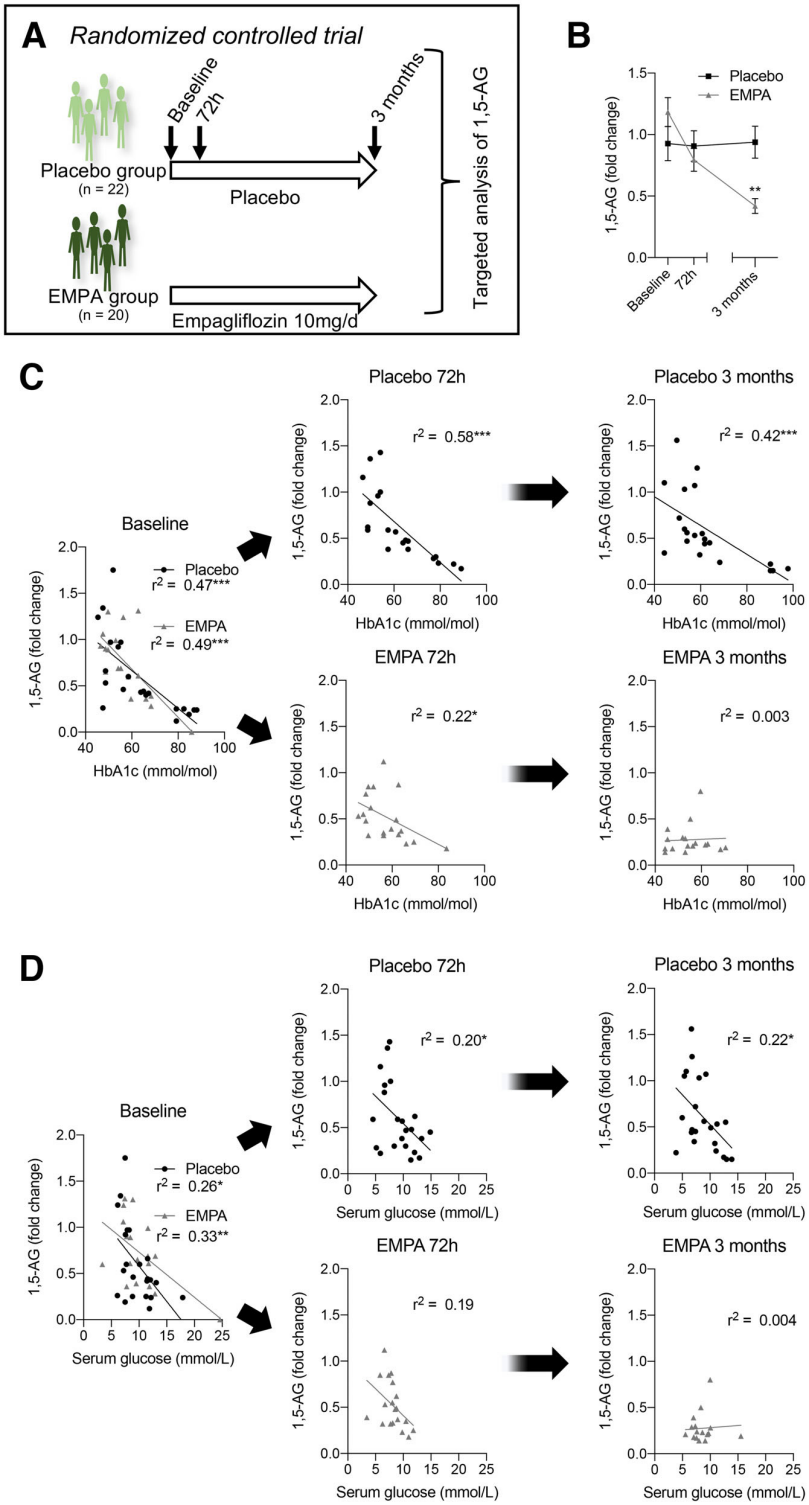
Dependency of 1,5‐anhydroglucitol on SGTL2 is confirmed in a placebo‑controlled, randomized, double blind human trial with empagliflozin. (A) Targeted serum analysis of 1,5‐anhydroglucitol (1,5‐AG) was performed in a in a placebo‑controlled, randomized, double blind human trial with empagliflozin at baseline, 72 h, and 3 months after treatment. Placebo group: n = 22; empagliflozin (10 mg once daily)(EMPA): n = 20. (B) Serum levels of 1,5‐AG over time in placebo‐ and empagliflozin‐treated patients. **P < 0.01 by mixed‐effects analysis with Sidak's multiple comparison test. (C) Pearson correlation analysis of 1,5‐AG and HbA1c at baseline, 72 h, and 3 months after placebo/empagliflozin treatment. *P < 0.05, ***P < 0.001. (D) Pearson correlation analysis of 1,5‐AG and serum glucose at baseline, 72 h, and 3 months after placebo/empagliflozin treatment. *P < 0.05, **P < 0.01, ***P < 0.001

In this manuscript, we used three different metabolomic data sets to study biomarkers and substrate utilization in relation to diabetes and glycemic control. Comparing human and mouse metabolomics, we found BCAA, glutathione and acylcarnitine metabolism as well as 3‐hydroxy fatty acids to be increased in humans and mice with diabetes (discussed in the Supporting Information). In addition, 1,5‐AG, a metabolite that has earlier been recognized as glycemic marker,^4^, ^5^ was found to be downregulated in an diabetic environment while further reduced by SGLT2 inhibition.

1,5‐AG is structurally similar to glucose and has been recognized as short‐ to mid‐term marker of glycemic control in several studies.^4^, ^5^ In this comprehensive, unbiased metabolomics approach, 1,5‐AG proved as the most reliable predictor for poor glycemic control. Circulating 1,5‐AG is mainly derived from food, little metabolized and excreted unaltered to the urine. Under normoglycemic conditions, 1,5‐AG is subjected to renal filtration and complete re‐absorption in the proximal tubules. Under hyperglycemic conditions with glucosuria, glucose competitively inhibits the re‐absorption of 1,5‐AG with concomitant urinary excretion resulting in decreased 1,5‐AG serum levels.^6^ Yamanouchi et al. found that 1,5‐AG competitively inhibited re‐absorption of fructose and mannose.^7^ Therefore, re‐absorption of 1,5‐AG in the kidney must be facilitated by a transporter specific for these sugars and glucose. Tazawa et al. suggested SGLT4 to be the main transporter for 1,5‐AG.^8^ We discovered SGLT2 inhibition to reduce 1,5‐AG levels possibly by increasing urinary glucose excretion. Our data suggest that the highly selective SGLT2 inhibitor empagliflozin (>3500‐fold selectivity for SGLT2 over SGLT4) strongly diminishes serum levels of 1,5‐AG, likewise in humans and mice. Therefore, our data indicate strong relevance of SGLT2 for 1,5‐AG reabsorption. A decrease of 1,5‐AG has also been observed after treatment with SGLT2 inhibitor canagliflozin.^9^ Wortman et al treated nondiabetic patients suffering from glycogen storage disease type Ib with empagliflozin. Empagliflozin reduced levels of 1,5‐AG and prevented formation of 1,5‐AG‐6‐phosphate, which has been suspected to cause neutropenia in these patients.^10^

In conclusion, our translational approach gives novel insights into the metabolic profile associated with T2D. Particularly, our unbiased metabolomics identified 1,5‐AG as an excellent biomarker for glycemic control in humans and mice. We further demonstrate SGLT2 inhibition to lower 1,5‐AG serum levels, most likely mediated by reduced renal reabsorption. 1,5‐AG should consequently not be used as an indicator of glycemic control in patients with SGLT2 inhibitor treatment.

## ETHICS APPROVAL AND CONSENT TO PARTICIPATE

All subjects gave written informed consent. Ethics of myocardial infarction cohort were approved by the ethical committee of Policlinico Tor Vergata University of Rome. Ethics of Empagliflozin registry cohort were approved by the ethics committee of the Medical Faculty of RWTH Aachen University. Ethics of the placebo‑controlled, randomized, double blind human trial with empagliflozin were approved by the ethics committee of the Medical Faculty of RWTH Aachen University and the German Federal Institute for Drugs and Medical Devices (Bundesinstitut für Arzneimittel und Medizinprodukte, BfArM).

Animal experiments were approved by the government of North Rhine‐Westphalia, Germany.

## AVAILABILITY OF DATA AND MATERIALS

The datasets used and analyzed during the current study are available from the corresponding author on reasonable request.

## CONFLICT OF INTEREST

N.M. has received support for clinical trial leadership from Boehringer Ingelheim, Novo Nordisk, served as a consultant to Boehringer Ingelheim, Merck, Novo Nordisk, AstraZeneca, BMS, received grant support from Boehringer Ingelheim, Merck, Novo Nordisk, and served as a speaker for Boehringer Ingelheim, Merck, Novo Nordisk, Lilly, BMS, and Astra Zeneca. N.M. declines all personal compensation from pharma or device companies.

K.S. served as a consultant to Amgen, Boehringer Ingelheim, AstraZeneca, Lilly, received grant support from Boehringer Ingelheim, and served as a speaker for Amgen, AstraZeneca, Boehringer Ingelheim, Lilly, MSD, Novo Nordisk, Novartis, OmniaMed.

M.F. served as a consultant for Boehringer Ingelheim, Merck Sharpe&Dohme, Lilly & Co, Janssen, Sanofi, Amgen.

M.L. received grants and personal fees from Boehringer Ingelheim, MSD and Novo Nordisk, personal fees from Amgen, Sanofi, Astra Zeneca, Bayer and Lilly.

## FUNDING INFORMATION

B.A.K. was supported by a grants from the Deutsche Stiftung für Herzforschung (DSHF)[F‐43‐16] and RWTH Aachen University (START); N.M. and M.L. were supported by the Deutsche Forschungsgemeinschaft (German Research Foundation; SFB/TRR 219; Project‐ID 322900939 [M03, M05]. M.L. was supported by the Interreg V‐A grant EURlipids. K.S. was supported by the Deutsche Forschungsgemeinschaft (German Research Foundation; SFB/TRR219 C‐07). K.S., N.M. and M.L. were further supported by a CORONA Foundation grant. M.F. laboratory was in part funded by EU‐FP7 FLORINASH [grant agreement ID: 241913], Ministry of University (MIUR) Progetti di Ricerca di Interesse Nazionale (PRIN) [protocol number 2015MPESJS_004 and 2017FM74HK], Fondazione Roma call for Non‐Communicable Diseases NCD 2014, EU‐FP7 EURHYTHDIA [grant agreement ID: 278397]. The funders were not involved in the design of the study; the collection, analysis, and interpretation of data; writing the report; and did not impose any restrictions regarding the publication of the report.

## AUTHOR CONTRIBUTIONS

Conceptualization, B.A.K., M.F., N.M., and M.L.; Methodology, B.A.K., M.F., and M.L.; Development or design of methodology, B.A.K., M.F., and M.L.; Software, B.A.K.; Formal Analysis, B.A.K., M.F., and M.L.; Investigation, B.A.K., J.M., K.T., M.R. A.A., J.A., B.G., K.S., F.R., R.S.; Untargeted metabolomics analysis, A.A., J.A.; Resources, B.A.K., M.F., and M.L.; Data Curation, B.A.K.; Writing – Original Draft, B.A.K.; Writing – Review & Editing, B.A.K., M.F., K.S., N.M., and M.L.; Visualization, B.A.K.; Supervision, M.F., N.M., and M.L.; Project Administration, B.A.K., M.F., N.M., and M.L.; Funding Acquisition, B.A.K, K.S., N.M., M.F., and M.L.

## Supporting information

Supporting Information

Supporting Information

Supporting Information

Supporting Information
