# Supplementary material for: Human and mouse non‐targeted metabolomics identify 1,5‐anhydroglucitol as SGLT2‐dependent glycemic marker
Source: Clin Transl Med. 2021 Jun 27;11(6):e470. doi: 10.1002/ctm2.470 (PMC8236115; doi:10.1002/ctm2.470)
Supplement: Supplementary file 1 — Supporting Information [file CTM2-11-e470-s001.pdf]

Supplementary Figure S1

Myocardial infarction cohort

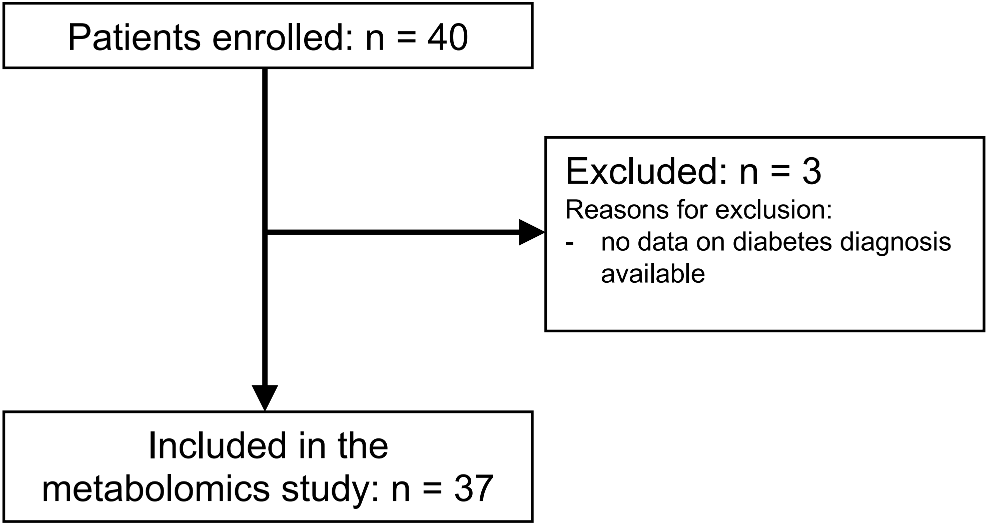

Supplementary Figure S1: STROBE diagram of myocardial infarction cohort.

Supplementary Figure S2

Empagliflozin registry

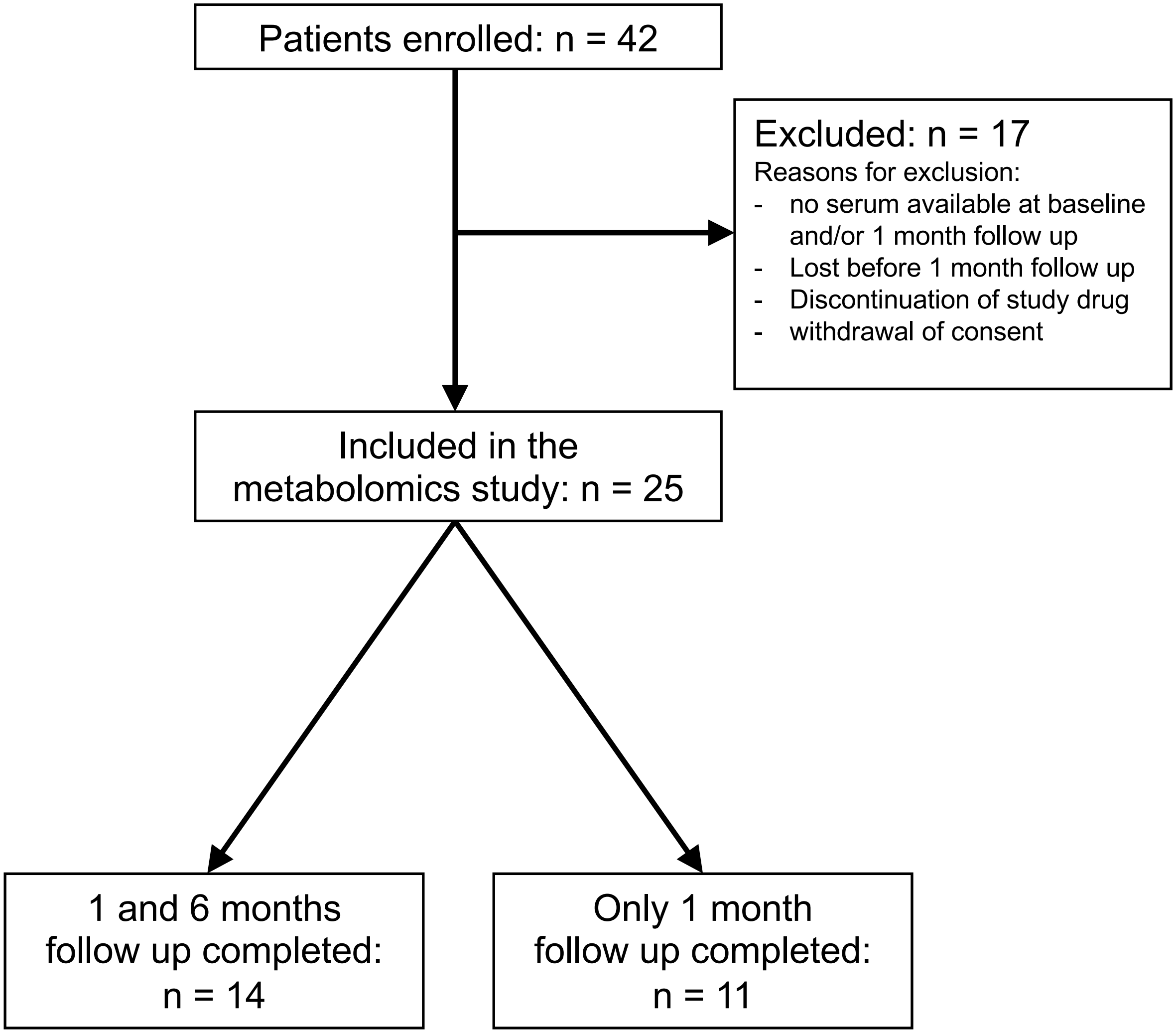

Supplementary Figure S2: STROBE diagram of the Empagliflozin registry.

Supplementary Figure S3

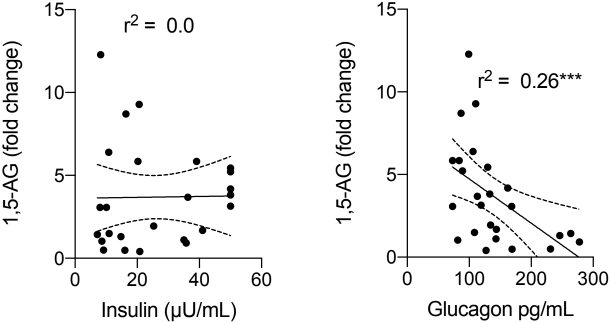

Supplementary Figure S3:
1,5-anhydroglucitol (1,5-AG) correlates with glucagon, but not with insulin in diabetic patients. Pearson correlation: r^2. ***P < 0.001
