# Supplementary material for: Human and mouse non‐targeted metabolomics identify 1,5‐anhydroglucitol as SGLT2‐dependent glycemic marker
Source: Clin Transl Med. 2021 Jun 27;11(6):e470. doi: 10.1002/ctm2.470 (PMC8236115; doi:10.1002/ctm2.470)
Supplement: Supplementary file 2 — Supporting Information [file CTM2-11-e470-s003.pdf]

SUPPLEMENTARY TABLES S1-S8

Supplementary Table S1: Baseline characteristics of human myocardial infarction cohort.

Parameter	Ctrl	T2D	P-value
Number	30	7	
Age, years	59.5 ± 11.2	61.4 ± 11.5	0.692
Gender, % male	100.0	100.0	0.999
Obesity, %	13.3	14.2	0.999
Hypertension, %	60.0	71.4	0.687
Dyslipidemia, %	56.7	71.4	0.677
Smoker, %	40.0	42.9	0.999
eGFR, mL/min/1.73m^2	84.1 ± 19.5	65.6 ± 20.2	**0.031**
Serum glucose, mmol/L	6.8 ± 1.6	12.9 ± 7.3	**0.002**
Cholesterol, total, mmol/L	4.6 ± 1.1	4.0 ± 1.1	0.142
Cholesterol, HDL, mmol/L	1.1 ± 0.2	1.0 ± 0.3	0.376
Cholesterol, LDL, mmol/L	3.2 ± 0.9	2.5 ± 0.6	0.061
Triglycerides, mmol/L	1.5 ± 0.7	1.7 ± 1.3	0.546

Supplementary Table S1 legend:

Baseline characteristics of patients hospitalized for myocardial infarction with type 2 diabetes mellitus (T2D, n = 7) and without diabetes (Ctrl, n = 30). Analysis by two-sided t-test or Chi-square test for categorical variables. Data are the mean ± S.D. Bold values indicate a significance with P < 0.05.

Supplementary Table S2: Baseline characteristics of human Empagliflozin Registry.

Parameter	T1	T2	T3	P-value
Number	9	7	9	
Age, years	61.4 ± 11.0	67.4 ± 13.5	62.0 ± 5.6	0.470
BMI, kg/m^2	30.0 ± 5.2	33.4 ± 5.8	31.8 ± 4.1	0.481
Diabetes duration, years	8.7 ± 6.3	16.0 ± 5.1	9.3 ± 3.2	**0.030**
HbA1c, mmol/mol	56.6 ± 2.0	66.9 ± 4.0	83.2 ± 10.6	**<0.001**
Serum Glucose, mmol/L	8.5 ± 0.6	11.1 ± 1.4	13.7 ± 1.7	**<0.001**
Insulin, µU/mL	28.1 ± 18.9	26.8 ± 16.1	22.7 ± 14.5	0.780
Glucagon, pg/mL	95.6 ± 19.7	144.4 ± 57.2	178.2 ± 60.7	**0.006**
eGFR, $mL/min/1.73m^2$	88.2 ± 9.3	81.5 ± 20.0	68.2 ± 13.2	**0.022**
HDL cholesterol, mmol/L	1.1 ± 0.2	1.0 ± 0.2	0.9 ± 0.4	0.149
LDL cholesterol, mmol/L	2.7 ± 0.8	2.4 ± 1.2	2.8 ± 2.0	0.828
Smoker, %	77.8	85.7	55.6	0.884
Hypertension, %	44.4	85.7	88.9	0.624
CVD, %	88.9	100.0	100.0	0.981
Previous myocardial infarction, %	66.7	57.1	55.5	0.968
Revascularization (CABG/PCI), %	66.7	100.0	88.9	0.855
Antiplatelet therapy, %	88.9	100.0	88.9	0.655
Anticoagulation, %	22.2	14.3	66.7	0.054
AT1/ACE inhibitors, %	77.8	71.4	100.0	0.247
Betablockers, %	77.8	100.0	66.7	0.249
Statins, %	88.9	100.0	88.9	0.655
Diuretics, %	55.6	71.4	66.7	0.474
Metformin, %	44.4	42.9	66.7	0.545
Glitazones, %	11.1	0.0	0.0	0.396
Sulfonylurea, %	0.0	0.0	22.2	0.149
DPPIV inhibitors, %	22.2	14.3	44.4	0.366
Insulin therapy, %	55.6	28.6	44.4	0.559

Supplementary Table S2 legend:

Baseline characteristics of patients with type 2 diabetes mellitus included in the Empagliflozin Registry at baseline (before treatment with empagliflozin). Groups divided in tertiles on basis of HbA1c: tertile 1 (T1, n = 9): HbA1c <60mmol/mol; tertile 2 (T2, n = 7): HbA1c 60-72mmol/mol; tertile 3 (T3, n = 9): HbA1c >72mmol/mol. Analysis by 1-way ANOVA or Chi-square test for categorical variables. Data are the mean ± S.D. Bold values indicate a significance with $P < 0.05$.

Supplementary Table S3 | Metabolites associated to type 2 diabetes in the myocardial infarction cohort.
Patients with type 2 diabetes mellitus: n = 7, control patients: n = 30. Analysis by unpaired two-sided t-test corrected for multiple testing by false discovery approach (FDR). "X-" indicate unidentified metabolites.

	t-stat	P-value	minusLOG10(p)	FDR
X - 12104	4,79	0,00003	4,53	0,015
1,5-anhydroglucitol (1,5-AG)	-4,09	0,00024	3,62	0,055
bilirubin E,Z or Z,E	3,98	0,00033	3,48	0,055
X - 16534	3,76	0,00063	3,20	0,071
3-hydroxybutyrylcarnitine 1	3,72	0,00070	3,15	0,071
X - 12122	3,50	0,00130	2,89	0,108
X - 11727	3,39	0,00177	2,75	0,127
X - 12849	3,29	0,00227	2,64	0,132
C-glycosyltryptophan	3,26	0,00246	2,61	0,132
X - 21207	3,21	0,00281	2,55	0,132
X - 12100	3,19	0,00301	2,52	0,132
X - 17191	3,17	0,00316	2,50	0,132
X - 17184	3,10	0,00385	2,41	0,146
X - 16129	3,07	0,00407	2,39	0,146
3-hydroxyisobutyrate	3,01	0,00487	2,31	0,147
beta-hydroxyisovaleroylcarnitine	2,99	0,00510	2,29	0,147
3-methylglutarylcarnitine C6	2,98	0,00518	2,29	0,147
heme	2,96	0,00552	2,26	0,147
cortisol	2,95	0,00564	2,25	0,147
X - 14384	2,90	0,00637	2,20	0,147
palmitoylcarnitine C16	2,90	0,00646	2,19	0,147
X - 16135	2,89	0,00662	2,18	0,147
oleoylcarnitine C181	2,87	0,00687	2,16	0,147
X - 21365	2,86	0,00702	2,15	0,147
glutathione, oxidized GSSG	2,82	0,00786	2,10	0,156
2-piperidinone	2,81	0,00807	2,09	0,156
octadecanedioate	2,79	0,00851	2,07	0,158
histidine	-2,76	0,00915	2,04	0,160
succinylcarnitine C4-DC	2,76	0,00925	2,03	0,160
X - 12775	2,69	0,01087	1,96	0,180
X - 17256	2,68	0,01115	1,95	0,180
4-acetamidobutanoate	2,65	0,01212	1,92	0,190
X - 15675	2,58	0,01410	1,85	0,208
N2,N2-dimethylguanosine	2,58	0,01427	1,85	0,208
imidazole propionate	2,57	0,01455	1,84	0,208
5alpha-pregnan-3beta,20alpha-diol disulfate	2,53	0,01592	1,80	0,218
X - 16134	2,53	0,01607	1,79	0,218
X - 17175	2,51	0,01704	1,77	0,218
homocitrulline	2,50	0,01732	1,76	0,218
1-palmitoyl-GPI 160	-2,49	0,01783	1,75	0,218
stearoylcarnitine C18	2,49	0,01786	1,75	0,218
X - 21223	2,45	0,01951	1,71	0,233
laurylcarnitine C12	2,44	0,01997	1,70	0,233
X - 16094	-2,41	0,02113	1,68	0,240
X - 13543	2,40	0,02175	1,66	0,240
acisoga	2,40	0,02201	1,66	0,240
X - 11538	2,37	0,02328	1,63	0,248
urea	2,34	0,02521	1,60	0,255
nicotinamide	2,34	0,02527	1,60	0,255
xanthine	2,33	0,02546	1,59	0,255
3-hydroxybutyrate BHBA	2,30	0,02753	1,56	0,270
X - 18929	2,28	0,02851	1,55	0,273
N6-acetyllysine	2,26	0,03043	1,52	0,273
X - 15306	-2,26	0,03049	1,52	0,273
N-oleoyltaurine	2,25	0,03055	1,52	0,273

N-acetylserine	2,25	0,03075	1,51	0,273
ergothioneine	2,24	0,03157	1,50	0,273
X - 11491	2,23	0,03254	1,49	0,273
alpha-hydroxyisovalerate	2,22	0,03282	1,48	0,273
aspartate	2,22	0,03314	1,48	0,273
X - 11334	2,22	0,03329	1,48	0,273
N-acetyl-1-methylhistidine	2,19	0,03552	1,45	0,273
X - 18223	-2,19	0,03568	1,45	0,273
X - 19900	2,18	0,03627	1,44	0,273
allopurinol riboside	2,17	0,03649	1,44	0,273
acetylcarnitine C2	2,17	0,03661	1,44	0,273
cis-aconitate	2,17	0,03689	1,43	0,273
1-stearoyl-GPI 180	-2,16	0,03732	1,43	0,273
N6-carbamoylthreonyladenosine	2,16	0,03757	1,43	0,273
X - 16133	2,16	0,03812	1,42	0,273
N1-methylguanosine	2,13	0,03996	1,40	0,280
cysteine-glutathione disulfide	2,13	0,04059	1,39	0,280
X - 12850	2,12	0,04073	1,39	0,280
X - 12855	2,11	0,04230	1,37	0,285
X - 20006	2,10	0,04268	1,37	0,285

Supplementary Table S4 | Metabolites associated to a diabetic phenotype in a Db/Db mouse model of diabetes. Diabetic Db/Db mice n = 6, non-diabetic Db/+ mice n = 6. Analysis by unpaired two-sided t-test corrected for multiple testing by false discovery approach (FDR). "X-" indicate unidentified metabolites.

	**t-stat**	**P-value**	**minusLOG10(p)**	**FDR**
palmitoyl ethanolamide	8,58	0,00001	5,20	0,003
gamma-glutamylleucine	8,46	0,00001	5,14	0,003
1,5-anhydroglucitol (1,5-AG)	-7,96	0,00001	4,91	0,003
glucose	7,43	0,00002	4,65	0,004
arachidonate 204n6	7,09	0,00003	4,48	0,004
N-oleoyltaurine	7,02	0,00004	4,44	0,004
indoleacetate	6,68	0,00006	4,26	0,006
flavin adenine dinucleotide FAD	6,04	0,00012	3,90	0,010
N-palmitoyltaurine	6,02	0,00013	3,89	0,010
eicosapentaenoate EPA 205n3	5,88	0,00015	3,81	0,010
gamma-glutamylisoleucine	5,85	0,00016	3,79	0,010
3-hydroxyoctanoate	5,71	0,00020	3,71	0,010
trans-4-hydroxyproline	-5,69	0,00020	3,70	0,010
folate	-5,69	0,00020	3,70	0,010
S-methylcysteine	5,60	0,00023	3,64	0,011
oleoyl ethanolamide	5,41	0,00030	3,53	0,013
3-hydroxyhexanoate	5,31	0,00034	3,46	0,015
ergothioneine	5,20	0,00040	3,40	0,016
1-stearoyl-GPG 180	5,17	0,00042	3,38	0,016
2-hydroxystearate	5,05	0,00050	3,30	0,018
dihomo-linolenate 203n3 or n6	5,04	0,00051	3,29	0,018
phenylalanine	4,92	0,00061	3,22	0,020
taurocholate	4,88	0,00064	3,19	0,020
X - 21353	4,60	0,00098	3,01	0,030
X - 12524	4,52	0,00111	2,96	0,032
beta-muricholate	-4,47	0,00120	2,92	0,034
docosapentaenoate n3 DPA 225n3	4,43	0,00128	2,89	0,035
X - 24548	4,38	0,00137	2,86	0,035
X - 15472	4,37	0,00140	2,85	0,035
tetradecadienoate 142	4,23	0,00174	2,76	0,042
X - 22764	-4,21	0,00179	2,75	0,042
docosahexaenoate DHA 226n3	4,15	0,00198	2,70	0,045
deoxycholate	4,10	0,00215	2,67	0,048
gamma-glutamyl-2-aminobutyrate	3,97	0,00262	2,58	0,055
docosatrienoate 223n3	3,94	0,00276	2,56	0,055
ornithine	3,93	0,00283	2,55	0,055
indoleacrylate	3,89	0,00302	2,52	0,055
X - 23369	3,89	0,00303	2,52	0,055
tauro-beta-muricholate	3,87	0,00311	2,51	0,055
tauroursodeoxycholate	3,87	0,00313	2,50	0,055
sphinganine	3,86	0,00315	2,50	0,055
linoleoyl ethanolamide	3,86	0,00316	2,50	0,055
taurodeoxycholate	3,81	0,00343	2,46	0,058
5-methyluridine ribothymidine	3,79	0,00353	2,45	0,059
X - 11315	3,78	0,00361	2,44	0,059
S-methylglutathione	3,75	0,00378	2,42	0,060
X - 24748	3,71	0,00403	2,39	0,063
N-acetyltryptophan	3,60	0,00486	2,31	0,072
uridine	3,59	0,00497	2,30	0,072

1-carboxyethylleucine	3,58	0,00498	2,30	0,072
X - 18913	3,57	0,00513	2,29	0,072
X - 16087	3,56	0,00516	2,29	0,072
3-hydroxyhexanoylcarnitine 1	3,50	0,00575	2,24	0,077
3-formylindole	3,49	0,00579	2,24	0,077
cysteine-glutathione disulfide	3,49	0,00584	2,23	0,077
taurohyodeoxycholic acid	3,47	0,00599	2,22	0,078
N-methylproline	3,42	0,00656	2,18	0,082
5-methylcytidine	-3,41	0,00663	2,18	0,082
2-stearoyl-GPE 180	3,41	0,00669	2,17	0,082
2-hydroxyarachidate	3,40	0,00673	2,17	0,082
methyl-4-hydroxybenzoate	3,39	0,00684	2,17	0,082
tryptophan	3,32	0,00781	2,11	0,092
dihomo-linoleate 202n6	3,26	0,00864	2,06	0,099
X - 17620	3,25	0,00871	2,06	0,099
2-hydroxypalmitate	3,23	0,00904	2,04	0,100
creatine	3,22	0,00918	2,04	0,100
guanosine	-3,21	0,00927	2,03	0,100
X - 21342	3,21	0,00940	2,03	0,100
glu-gly-asn-val	3,19	0,00972	2,01	0,100
taurochenodeoxycholate	3,18	0,00976	2,01	0,100
1-stearoyl-GPI 180	3,18	0,00979	2,01	0,100
3-hydroxyoleate	3,18	0,00988	2,01	0,100
2-hydroxydecanoate	3,16	0,01013	1,99	0,100
gamma-glutamyl-epsilon-lysine	-3,16	0,01019	1,99	0,100
2-aminoheptanoate	3,16	0,01024	1,99	0,100
kynurenine	3,13	0,01070	1,97	0,103
X - 24665	3,12	0,01096	1,96	0,104
7-ketodeoxycholate	-3,11	0,01113	1,95	0,104
X - 21362	3,09	0,01147	1,94	0,106
guanosine 5'- monophosphate 5'-GMP	-3,05	0,01226	1,91	0,109
oleoylcarnitine C181	3,05	0,01235	1,91	0,109
isoleucine	3,04	0,01237	1,91	0,109
dodecanedioate C12-DC	3,04	0,01239	1,91	0,109
cytidine	3,03	0,01257	1,90	0,109
linolenate alpha or gamma 183n3 or 6	3,01	0,01314	1,88	0,112
thymidine	-3,00	0,01329	1,88	0,112
alanylleucine	3,00	0,01335	1,87	0,112
3-hydroxyindolin-2-one	2,98	0,01382	1,86	0,114
2-palmitoyl-GPC 160	2,96	0,01425	1,85	0,117
N-stearoyltaurine	2,94	0,01477	1,83	0,119
pyruvate	2,94	0,01488	1,83	0,119
arginine	-2,92	0,01519	1,82	0,120
X - 18922	2,91	0,01569	1,80	0,123
indolin-2-one	2,89	0,01620	1,79	0,126
choline	-2,87	0,01659	1,78	0,127
phenylacetate	2,84	0,01743	1,76	0,132
N-acetyl-2-aminooctanoate	2,83	0,01796	1,75	0,134
acetylcarnitine C2	2,82	0,01806	1,74	0,134
gamma-glutamylvaline	2,82	0,01816	1,74	0,134
phenylacetylcarnitine	2,80	0,01893	1,72	0,138
3-methylhistidine	-2,79	0,01906	1,72	0,138
ophthalmate	2,76	0,02000	1,70	0,142
1-stearoyl-GPE 180	2,76	0,02004	1,70	0,142
methionine sulfoxide	-2,73	0,02108	1,68	0,148

2-hydroxyoctanoate	2,71	0,02178	1,66	0,151
benzoylcarnitine	-2,68	0,02295	1,64	0,157
myristoleoylcarnitine C141	2,68	0,02300	1,64	0,157
3-hydroxydecanoate	2,68	0,02319	1,63	0,157
10-undecenoate 111n1	-2,67	0,02342	1,63	0,157
leucine	2,62	0,02539	1,60	0,168
stearate 180	2,62	0,02554	1,59	0,168
1-palmitoyl-GPG 160	2,60	0,02666	1,57	0,173
isobutyrylglycine	2,59	0,02688	1,57	0,173
1-carboxyethylvaline	2,59	0,02700	1,57	0,173
2-linoleoylglycerol 182	2,57	0,02766	1,56	0,175
palmitamide 160	2,56	0,02832	1,55	0,178
gamma-glutamylphenylalanine	2,55	0,02870	1,54	0,179
2-hydroxy-3-methylvalerate	2,52	0,03033	1,52	0,187
3-hydroxylaurate	2,50	0,03160	1,50	0,191
adrenate 224n6	2,50	0,03165	1,50	0,191
1-palmitoyl-GPE 160	2,49	0,03190	1,50	0,191
glycylvaline	2,49	0,03191	1,50	0,191
X - 12953	2,47	0,03286	1,48	0,195
heneicosapentaenoate 215n3	2,44	0,03502	1,46	0,206
3beta-hydroxy-5-cholestenoate	2,42	0,03605	1,44	0,210
methyl-4-hydroxybenzoate sulfate	2,41	0,03648	1,44	0,211
3-phenylpropionate hydrocinnamate	2,39	0,03797	1,42	0,218
valine	2,38	0,03833	1,42	0,218
2'-deoxyuridine	-2,37	0,03902	1,41	0,219
1-docosahexaenoylglycerol 226	2,37	0,03906	1,41	0,219
alpha-CEHC	-2,37	0,03952	1,40	0,220
11-dehydrocorticosterone	2,36	0,03985	1,40	0,220
1-linoleoyl-GPG 182	2,35	0,04033	1,39	0,221
2-oleoylglycerol 181	2,34	0,04131	1,38	0,225
8-hydroxyoctanoate	-2,32	0,04242	1,37	0,229
oleamide	2,32	0,04265	1,37	0,229
phenylpyruvate	2,29	0,04488	1,35	0,238
12-HHTrE	2,29	0,04507	1,35	0,238
uracil	2,28	0,04592	1,34	0,241
3-methoxytyrosine	-2,26	0,04733	1,32	0,246
glutathione, oxidized GSSG	2,25	0,04823	1,32	0,249
1-arachidonoyl-GPE 204n6	2,24	0,04864	1,31	0,249
docosatrienoate 223n6	2,24	0,04886	1,31	0,249
glutathione, reduced GSH	2,23	0,04976	1,30	0,252

Supplementary Table S5 | Multivariate analysis identifies 1,5-anhydroglucitol (1,5-AG) as biomarker for diabetes independently of baseline characteristics. Analysis in the myocardial infarction (MI) cohort. Patients with type 2 diabetes mellitus: n = 7, control patients: n = 30. Significant P-values are bold. Type of MI: ST- or non-ST-elevation MI. GFR: glomerular filtration rate.

Dependent Variable: Group (No diabetes versus diabetes)

	Unstandardized Coefficients		Standardized Coefficients		
	B	Std. Error	Beta	t	Significance
(Constant)	1,472	0,386		3,817	0,001
1,5-AG	-0,416	0,127	-0,493	-3,289	**0,003**
Type of MI	-0,185	0,118	-0,237	-1,569	0,129
Total cholesterol	0,008	0,009	0,808	0,856	0,400
Cholesterol, LDL	-0,011	0,010	-0,916	-1,158	0,257
Cholesterol, HDL	-0,004	0,011	-0,107	-0,388	0,701
Triglycerides	-0,001	0,002	-0,243	-0,787	0,438
GFR	-0,006	0,003	-0,334	-2,114	0,044
Smoker	0,199	0,126	0,250	1,579	0,126
Hypertension	-0,015	0,122	-0,018	-0,119	0,906
Obesity	0,034	0,170	0,030	0,200	0,843

Supplementary Table S6 | Metabolites associated to glycated hemoglobin (HbA1c) in the Empagliflozin registry. n = 25. Pearson correlation corrected for multiple testing by false discovery approach (FDR). "X-" indicate unidentified metabolites.

	r	t-stat	P-value	FDR
3-hydroxysebacate	0,66	4,16550	0,00037	0,115
3-hydroxydecanoate	0,65	4,13300	0,00040	0,115
3-hydroxyoctanoate	0,65	4,10590	0,00043	0,115
HEPES	0,65	4,08560	0,00045	0,115
1,5-anhydroglucitol 1,5-AG	-0,64	-3,99640	0,00057	0,119
X - 14939	0,62	3,79460	0,00094	0,169
X - 18938	0,60	3,64000	0,00137	0,203
X - 16570	0,60	3,57960	0,00159	0,203
X - 17654	0,60	3,57450	0,00161	0,203
alliin	0,59	3,47360	0,00206	0,222
X - 21319	0,59	3,46290	0,00211	0,222
X - 21339	0,58	3,40520	0,00243	0,230
X - 12812	0,58	3,38380	0,00256	0,230
lanthionine	0,56	3,27700	0,00331	0,278
1-linolenoyl-GPC 183	0,55	3,15020	0,00448	0,338
X - 21353	0,55	3,14300	0,00456	0,338
dodecanedioate	0,54	3,10880	0,00494	0,339
X - 19561	0,54	3,09570	0,00510	0,339
6-oxopiperidine-2-carboxylate	0,53	3,03680	0,00586	0,370
X - 11549	0,53	3,01060	0,00623	0,373
3-hydroxyhexanoate	0,53	2,99260	0,00650	0,373
X - 11478	0,52	2,95670	0,00707	0,378
X - 15486	0,52	2,94980	0,00719	0,378
androstenediol 3beta,17beta disulfate 1	0,52	2,90600	0,00796	0,378
X - 16947	0,52	2,88830	0,00829	0,378
X - 21849	0,51	2,87410	0,00857	0,378
3-hydroxylaurate	0,51	2,86430	0,00877	0,378
X - 21845	0,51	2,86220	0,00881	0,378
ADSGEGDFXAEGGGVR	0,51	2,85320	0,00900	0,378
eicosanodioate	0,51	2,83110	0,00947	0,385
X - 11308	0,50	2,79340	0,01033	0,407
pantothenate	0,50	2,76120	0,01112	0,410
X - 17653	0,50	2,75690	0,01123	0,410
N-formylanthranilic acid	0,50	2,74260	0,01160	0,410
X - 11261	0,50	2,73910	0,01169	0,410
alpha-hydroxymetoprolol	0,49	2,70930	0,01251	0,427
choline phosphate	0,49	2,69280	0,01299	0,431
N-acetylalliin	0,48	2,63360	0,01485	0,462
X - 22102	0,48	2,62980	0,01497	0,462
3beta,7alpha-dihydroxy-5-cholestenoate	0,48	2,61900	0,01534	0,462
X - 12830	0,48	2,61820	0,01537	0,462
X - 18889	0,47	2,53840	0,01837	0,525
phosphate	0,47	2,53230	0,01861	0,525
androstenediol 3beta,17beta monosulfate 1	0,47	2,52050	0,01911	0,525
X - 23294	-0,47	-2,52000	0,01913	0,525
21-hydroxypregnenolone disulfate	0,46	2,46670	0,02151	0,574
1-oleoyl-GPE 181	0,46	2,46020	0,02182	0,574
metoprolol acid metabolite	0,45	2,44360	0,02263	0,583
dihomo-linoleoylcarnitine C202	0,45	2,42950	0,02334	0,589
dehydroisoandrosterone sulfate DHEA-S	0,44	2,37880	0,02606	0,638
ximenoylcarnitine C261	0,44	2,37460	0,02629	0,638
metformin	0,44	2,36570	0,02680	0,638
X - 12462	0,44	2,34900	0,02779	0,649
X - 18901	0,44	2,32940	0,02898	0,656
etiocholanolone glucuronide	0,43	2,31490	0,02989	0,656
X - 18603	0,43	2,31470	0,02990	0,656
linolenoylcarnitine C183	0,43	2,30830	0,03032	0,656
4-hydroxycoumarin	0,43	2,30310	0,03066	0,656
pyridoxate	0,43	2,25860	0,03369	0,703
3-hydroxybutyrylcarnitine 2	0,43	2,25450	0,03399	0,703
X - 18921	0,42	2,23300	0,03557	0,724

androstenediol 3alpha, 17alpha monosulfate 3	0,42	2,22330	0,03631	0,727
sphingomyelin d182/242	-0,42	-2,20910	0,03740	0,738
xanthurenate	0,42	2,19490	0,03853	0,748
sphingomyelin d181/170, d171/180, d191/160	-0,41	-2,16910	0,04066	0,774
bradykinin, des-arg9	0,41	2,16380	0,04111	0,774
X - 22834	0,41	2,15580	0,04180	0,776
N-methyltaurine	0,41	2,14100	0,04310	0,778
phenylalanylglycine	-0,41	-2,14060	0,04314	0,778
X - 22764	0,40	2,11980	0,04503	0,800
glycodeoxycholate	0,40	2,09420	0,04746	0,815
X - 16935	0,40	2,08640	0,04823	0,815
X - 13737	0,40	2,08640	0,04823	0,815
X - 12798	-0,40	-2,08430	0,04843	0,815

Supplementary Table S7 | Metabolites associated to serum glucose in the Empagliflozin registry. n = 25. Pearson correlation corrected for multiple testing by false discovery approach (FDR). "X-" indicate unidentified metabolites.

	r	t-stat	P-value	FDR
X - 21849	0,69	4,51730	0,00016	0,076
X - 15486	0,66	4,23650	0,00031	0,076
linolenoylcarnitine C183	0,65	4,15360	0,00038	0,076
X - 21353	0,65	4,13890	0,00040	0,076
X - 14939	0,65	4,09340	0,00045	0,076
linoleoylcarnitine C182	0,64	4,02650	0,00053	0,076
dihomo-linoleoylcarnitine C202	0,64	4,02380	0,00053	0,076
X - 21319	0,64	4,01290	0,00054	0,076
X - 16570	0,62	3,81920	0,00088	0,107
X - 12101	0,62	3,79490	0,00093	0,107
X - 21339	0,61	3,71320	0,00114	0,120
3-hydroxybutyrylcarnitine 2	0,61	3,65320	0,00133	0,129
3-hydroxydecanoate	0,60	3,59370	0,00153	0,131
eicosanodioate	0,60	3,58600	0,00156	0,131
X - 11478	0,59	3,54290	0,00174	0,137
X - 11261	0,59	3,50490	0,00191	0,138
ximenoylcarnitine C261	0,59	3,48000	0,00202	0,138
X - 12063	0,59	3,46810	0,00208	0,138
pyridoxate	0,58	3,41540	0,00237	0,149
X - 17654	0,58	3,39400	0,00249	0,150
1-linoleoyl-GPI 182	0,57	3,33920	0,00285	0,163
X - 16480	0,57	3,31550	0,00302	0,165
1,5-anhydroglucitol 1,5-AG	-0,57	-3,28540	0,00324	0,165
X - 11852	0,56	3,28240	0,00327	0,165
3-hydroxyhexanoate	0,56	3,23290	0,00368	0,173
X - 21607	0,56	3,20120	0,00397	0,173
3-hydroxyoctanoate	0,55	3,18510	0,00412	0,173
X - 24527	0,55	3,18470	0,00413	0,173
3-hydroxysebacate	0,55	3,17470	0,00423	0,173
docosadioate	0,55	3,17360	0,00424	0,173
3-hydroxylaurate	0,55	3,13420	0,00465	0,181
androstenediol 3beta,17beta disulfate 1	0,55	3,12740	0,00473	0,181
X - 17705	0,54	3,11120	0,00492	0,182
dodecanedioate	0,54	3,08230	0,00526	0,188
2-aminoheptanoate	0,54	3,05470	0,00562	0,188
X - 11549	0,54	3,05410	0,00563	0,188
alliin	0,54	3,05230	0,00565	0,188
5alpha-androstan-3alpha,17alpha-diol disulfate	0,53	3,02510	0,00602	0,188
X - 18938	0,53	3,02360	0,00605	0,188
X - 11308	0,53	3,01970	0,00610	0,188
X - 16944	0,53	2,99950	0,00640	0,190
X - 12739	0,53	2,99460	0,00647	0,190
N-acetylalliin	0,52	2,95510	0,00710	0,204
malate	0,52	2,92990	0,00753	0,208
sphingomyelin d181/170, d171/180, d191/160	-0,52	-2,92780	0,00757	0,208
X - 18922	0,51	2,86190	0,00882	0,237
X - 22102	0,51	2,85290	0,00900	0,237
N4-acetylcytidine	0,51	2,83770	0,00932	0,239
lanthionine	0,51	2,83140	0,00946	0,239
glycodeoxycholate sulfate	0,50	2,79290	0,01034	0,251
sphingomyelin d181/202, d182/201, d161/222	-0,50	-2,79270	0,01034	0,251
X - 18603	0,50	2,78320	0,01057	0,251
X - 18889	0,50	2,76810	0,01094	0,251
alpha-CEHC sulfate	0,50	2,75390	0,01130	0,251
eicosenoylcarnitine C201	0,50	2,74900	0,01143	0,251
X - 17653	0,50	2,73650	0,01176	0,251
myristoleoylcarnitine C141	0,49	2,73180	0,01189	0,251
N1-Methyl-2-pyridone-5-carboxamide	0,49	2,72550	0,01206	0,251
adenosine 5'-monophosphate AMP	0,49	2,72220	0,01215	0,251
X - 24348	0,49	2,71820	0,01226	0,251
N-acetylmethionine sulfoxide	0,49	2,71630	0,01231	0,251
X - 12104	0,49	2,70190	0,01272	0,255

1-oleoyl-GPE 181	0,49	2,69470	0,01293	0,255
allantoin	0,49	2,67150	0,01363	0,260
1-linolenoyl-GPC 183	0,49	2,67030	0,01367	0,260
laurylcarnitine C12	0,49	2,66610	0,01380	0,260
arachidonoylcarnitine C204	0,48	2,65800	0,01405	0,260
X - 21467	0,48	2,65280	0,01422	0,260
X - 17327	0,48	2,63760	0,01472	0,260
X - 23756	0,48	2,62420	0,01516	0,260
betaine	0,48	2,62180	0,01525	0,260
gamma-CEHC glucuronide	0,48	2,61430	0,01550	0,260
HEPES	0,48	2,60600	0,01580	0,260
oleoylcarnitine C181	0,48	2,60580	0,01581	0,260
stearoylcarnitine C18	0,48	2,60510	0,01583	0,260
glucose	0,48	2,60390	0,01587	0,260
androstenediol 3beta,17beta disulfate 2	0,48	2,59270	0,01627	0,263
phosphate	0,47	2,57720	0,01685	0,269
palmitoleoylcarnitine C161	0,47	2,55230	0,01781	0,281
glycochenodeoxycholate glucuronide 1	0,47	2,53700	0,01842	0,286
X - 19561	0,47	2,53350	0,01857	0,286
X - 12830	0,47	2,52770	0,01881	0,286
X - 12442	0,46	2,51780	0,01922	0,289
X - 21845	0,46	2,49210	0,02035	0,289
X - 12456	0,46	2,49160	0,02037	0,289
sphingomyelin d182/210, d162/230	-0,46	-2,48530	0,02065	0,289
lignoceroylcarnitine C24	0,46	2,48300	0,02076	0,289
3beta,7alpha-dihydroxy-5-cholestenoate	0,46	2,48230	0,02079	0,289
X - 23294	-0,46	-2,48200	0,02081	0,289
xanthurenate	0,46	2,48060	0,02087	0,289
arachidate 200	0,46	2,46500	0,02160	0,293
N-alpha-acetylornithine	0,46	2,46270	0,02171	0,293
1-1-enyl-palmitoyl-2-oleoyl-GPE P-160/181	0,46	2,45980	0,02184	0,293
pantothenate	0,45	2,44450	0,02259	0,294
sphingomyelin d181/190, d191/180	-0,45	-2,44380	0,02262	0,294
X - 24949	0,45	2,44190	0,02271	0,294
gabapentin	0,45	2,43430	0,02310	0,294
X - 12462	0,45	2,42960	0,02333	0,294
stearate 180	0,45	2,42770	0,02343	0,294
glycocholenate sulfate	0,45	2,42590	0,02352	0,294
X - 11858	-0,45	-2,41130	0,02428	0,296
X - 21364	0,45	2,40330	0,02471	0,296
1-methylimidazoleacetate	0,45	2,40040	0,02486	0,296
nervonoylcarnitine C241	0,45	2,39820	0,02498	0,296
5alpha-pregnan-3beta,20alpha-diol disulfate	0,45	2,39550	0,02513	0,296
X - 17351	0,45	2,39180	0,02533	0,296
X - 13737	0,45	2,38320	0,02581	0,296
X - 22764	0,44	2,38120	0,02592	0,296
beta-citrylglutamate	0,44	2,37340	0,02636	0,296
erucate 221n9	0,44	2,36850	0,02664	0,296
aspartate	0,44	2,36260	0,02698	0,296
stearoyl sphingomyelin d181/180	-0,44	-2,35790	0,02726	0,296
X - 18921	0,44	2,35050	0,02770	0,296
2,3-dihydroxy-2-methylbutyrate	0,44	2,34910	0,02778	0,296
cytidine	0,44	2,34790	0,02785	0,296
5alpha-androstan-3beta,17beta-diol disulfate	0,44	2,34620	0,02795	0,296
X - 12472	0,44	2,34520	0,02801	0,296
adenosine 3',5'-cyclic monophosphate cAMP	0,44	2,33980	0,02834	0,296
X - 21821	0,44	2,33950	0,02836	0,296
17alpha-hydroxypregnanolone glucuronide	0,44	2,33870	0,02841	0,296
X - 14838	0,44	2,32660	0,02916	0,301
X - 11372	0,44	2,32380	0,02933	0,301
acesulfame	0,44	2,32060	0,02953	0,301
X - 23159	0,43	2,31640	0,02980	0,301
pregnen-diol disulfate C21H34O8S2	0,43	2,31150	0,03011	0,302
X - 11880	0,43	2,30780	0,03035	0,302
X - 22475	0,43	2,28160	0,03210	0,314

X - 01911	0,43	2,28150	0,03210	0,314
21-hydroxypregnenolone disulfate	0,43	2,26570	0,03319	0,322
bradykinin, des-arg9	0,43	2,26170	0,03348	0,323
glutamate	0,43	2,25350	0,03406	0,324
4-cholesten-3-one	0,42	2,25080	0,03426	0,324
X - 21343	0,42	2,24540	0,03465	0,324
1-1-enyl-oleoyl-GPE P-181	0,42	2,24530	0,03466	0,324
X - 24546	0,42	2,24020	0,03503	0,325
dihydroorotate	0,42	2,22250	0,03636	0,334
X - 24951	0,42	2,22110	0,03647	0,334
X - 12812	0,42	2,20940	0,03738	0,337
2-aminophenol sulfate	0,42	2,20850	0,03745	0,337
glycylvaline	0,42	2,20160	0,03799	0,337
S-allylcysteine	0,42	2,20020	0,03811	0,337
N-palmitoyl-sphingosine d181/160	0,42	2,19150	0,03880	0,337
N-formylmethionine	0,42	2,18810	0,03908	0,337
X - 12511	0,42	2,18790	0,03910	0,337
2-methylbutyrylcarnitine C5	0,41	2,18730	0,03914	0,337
L-urobilin	0,41	2,18340	0,03947	0,337
3-hydroxy-2-ethylpropionate	0,41	2,18030	0,03972	0,337
palmitoyl dihydrosphingomyelin d180/160	0,41	2,17720	0,03998	0,337
X - 13529	0,41	2,17600	0,04008	0,337
1-1-enyl-palmitoyl-GPE P-160	0,41	2,14520	0,04273	0,357
X - 18886	0,41	2,13920	0,04326	0,359
deoxycholate	0,41	2,13520	0,04362	0,360
X - 13835	0,41	2,12620	0,04444	0,363
X - 11440	0,40	2,12410	0,04463	0,363
5alpha-androstan-3alpha,17beta-diol disulfate	0,40	2,11370	0,04560	0,369
X - 24571	0,40	2,10730	0,04620	0,370
N-formylanthranilic acid	0,40	2,10580	0,04635	0,370
X - 24435	0,40	2,10160	0,04674	0,371
X - 21839	0,40	2,08490	0,04837	0,380
X - 24293	0,40	2,08360	0,04850	0,380
arachidonoylcholine	-0,40	-2,07010	0,04985	0,383
pipecolate	0,40	2,06990	0,04987	0,383

Supplementary Table S8 | Multivariate analysis shows correlation of 1,5-anhydroglucitol (1,5-AG) to glycated hemoglobin (HbA1c) independently of baseline characteristics in patients with type 2 diabetes. Empagliflozin registry, n = 25. Significant P-values are bold. CVD: cardiovascular disease. PPPIV: Dipeptidyl peptidase IV. GFR: glomerular filtration rate.

Dependent Variable: HbA1c

	Unstandardized Coefficients		Standardized Coefficients		
	B	Std. Error	Beta	t	Significance
(Constant)	75,210	50,288		1,496	0,178
1,5-AG	-2,683	1,131	-0,627	-2,372	**0,049**
Age	-0,269	0,460	-0,212	-0,584	0,578
Sex	-8,481	8,201	-0,266	-1,034	0,336
BMI	-0,362	0,710	-0,138	-0,510	0,626
Smoker	-5,982	8,846	-0,188	-0,676	0,521
Hypertension	3,722	8,144	0,130	0,457	0,662
CVD	24,643	15,936	0,383	1,546	0,166
Cholesterol, HDL	0,272	0,408	0,199	0,666	0,527
Cholesterol, LDL	0,054	0,061	0,210	0,895	0,401
GFR	-0,147	0,217	-0,183	-0,679	0,519
Metformin	10,733	6,625	0,408	1,620	0,149
Glitazones	-6,272	14,094	-0,097	-0,445	0,670
Sulfonylurea	6,503	10,512	0,140	0,619	0,556
DPPIV inhibitors	-0,349	7,273	-0,012	-0,048	0,963
Insulin therapy	6,662	6,503	0,253	1,025	0,340
