# Supplementary material for: Human and mouse non‐targeted metabolomics identify 1,5‐anhydroglucitol as SGLT2‐dependent glycemic marker
Source: Clin Transl Med. 2021 Jun 27;11(6):e470. doi: 10.1002/ctm2.470 (PMC8236115; doi:10.1002/ctm2.470)
Supplement: Supplementary file 3 — Supporting Information [file CTM2-11-e470-s002.pdf]

SUPPLEMENTARY DISCUSSION

Elevation of acylcarnitines in context of type 2 diabetes and poor glycemic control may indicate altered fatty utilization under these circumstances - as formation of acylcarnitines is required for fatty acid transportation into mitochondria for β-oxidation. In patients with type 2 diabetes, we found acylcarnitines to be related to increased glucose and HbA1c. Acylcarnitines are formed to transport fatty acids into mitochondria for β-oxidation. High serum concentrations may therefore indicate impaired fatty acid oxidation. Interestingly, circulating acylcarnitine levels have been shown to reflect the acylcarnitine profile of the heart, but not that of other tissues [1]. Our observations are consistent with other studies linking acylcarnitine concentrations with risk for type 2 diabetes [2, 3]. Direct causality is suggested by glucose intolerance and β-cell dysfunction of mice challenged with acylcarnitines [4, 5]. Serum levels of acylcarnitines have further been identified as biomarkers for coronary artery disease [6] and were predictive for cardiovascular events in patients with established cardiovascular disease (CVD) [7].

A notable finding in our study was the elevation in 3-hydroxy fatty acids in patients with type 2 diabetes and poor glycemic control. As 3-hydroxy fatty acids are recognized as catabolites of incomplete fatty acid oxidation this might be indicative for lipotoxicity and cellular stress in a poorly controlled metabolic environment [8]. Lipidomics have linked increased levels of 3-hydroxy acylcarnitines to mitochondrial dysfunction in diabetic rat myocardium, mediated through the enhancement of calcium-independent phospholipase A2 activity [9]. Accumulation of 3-hydroxylated fatty acids also occurs in genetic impairment of mitochondrial fatty acid oxidation (mitochondrial trifunctional protein and long-chain 3-hydroxyacyl-CoA dehydrogenase deficiency) leading to mitochondrial uncoupling, impaired cardiac energy homeostasis and cardiomyopathy [10]. Thus, 3-hydroxy fatty acids may not only be a indicator of defective metabolism in diabetes, but also propagate diabetic cardiomyopathy.

Metabolites of BCAA differed between non-diabetic and diabetic individuals in our study, but we found no evidence that BCAA are related to glycemic control in patients with established type 2 diabetes. A possible explanation could be that BCAA facilitate the development of diabetes, but do not relate to glycemic markers in advanced stages of the disease. Newgard et. al described a BCAA-related metabolic signature predicting insulin resistance in individuals without diabetes [11]. This finding is

consistent with other studies, which identified increased levels of BCAA as risk factor for insulin resistance [12, 13].

Interestingly, gut microbiota with enriched biosynthetic potential for BCAA synthesis have been linked to increased BCAA serum levels in individuals with insulin resistance [14]. There is emerging evidence that the gut microbiome plays a pivotal role in the development of cardiometabolic diseases, such as insulin resistance, type 2 diabetes and CVD. In part, the interactions between gut bacteria and host are mediated by gut-derived metabolites [15]. Consistently increased microbial metabolites in our Db/Db mouse models suggests modulation of the gut flora in diabetes, which agrees with previous reports [16]. Differences in gut-derived metabolites have however not been apparent in our human cohorts. This might be indicative for insufficient power of our study for these analysis or relate to diet and species differences.

BCAA and their metabolites have also positively been associated to CVD [17, 18]. BCAA metabolism might therefore contribute to the understanding of increased cardiovascular risk in patients with type 2 diabetes. Insulin resistance impairs BCAA metabolism in the heart [19], while defective BCAA catabolism has been show to promote heart failure [20]. In a previous study, we found that the SGLT2 inhibitor empagliflozin increases turn-over of BCAA in patients with type 2 diabetes [21]. Empagliflozin might therefor elicit cardioprotective properties via restoration of BCAA metabolism. Still we did not find a similar metabolic signature in empagliflozin treated diabetic Db/Db mice [22], Santos-Gallego et al. however observed empagliflozin to increase BCAA metabolism in a non-diabetic swine model of MI which was associated with improved outcome and reduced left ventricular remodeling [23]. These difference in response to SGLT2 inhibitors might relate to different experimental conditions and species.

REFERENCES

1. Makrecka-Kuka M, Sevostjanovs E, Vilks K, et al (2017) Plasma acylcarnitine concentrations reflect the acylcarnitine profile in cardiac tissues. Sci Rep 7:. https://doi.org/10.1038/s41598-017-17797-x
2. Sun L, Liang L, Gao X, et al (2016) Early prediction of developing type 2 diabetes by plasma acylcarnitines: A population-based study. Diabetes Care 39:1563–1570. https://doi.org/10.2337/dc16-0232
3. Guasch-Ferré M, Ruiz-Canela M, Li J, et al (2019) Plasma acylcarnitines and risk of type 2 diabetes in a mediterranean population at high cardiovascular risk. J Clin Endocrinol Metab 104:1508–1519. https://doi.org/10.1210/jc.2018-01000
4. Batchuluun B, Rijjal D Al, Prentice KJ, et al (2018) Elevated medium-chain acylcarnitines are associated with gestational diabetes mellitus and early progression to type 2 diabetes and induce pancreatic β-cell dysfunction. Diabetes 67:885–897. https://doi.org/10.2337/db17-1150
5. Aichler M, Borgmann D, Krumsiek J, et al (2017) N-acyl Taurines and Acylcarnitines Cause an Imbalance in Insulin Synthesis and Secretion Provoking β Cell Dysfunction in Type 2 Diabetes.

Cell Metab 25:1334-1347.e4. https://doi.org/10.1016/j.cmet.2017.04.012
6. Shah SH, Bain JR, Muehlbauer MJ, et al (2010) Association of a peripheral blood metabolic profile with coronary artery disease and risk of subsequent cardiovascular events. Circ Cardiovasc Genet 3:207–214. https://doi.org/10.1161/CIRCGENETICS.109.852814
7. Strand E, Pedersen ER, Svingen GFT, et al (2017) Serum acylcarnitines and risk of cardiovascular death and acute myocardial infarction in patients with stable angina pectoris. J Am Heart Assoc 6:. https://doi.org/10.1161/JAHA.116.003620
8. Jin SJ, Hoppel CL, Tserng KY (1992) Incomplete fatty acid oxidation. The production and epimerization of 3-hydroxy fatty acids. J Biol Chem 267:119–125. https://doi.org/10.1016/S0021-9258(18)48467-0
9. Su X, Han X, Mancuso DJ, et al (2005) Accumulation of long-chain acylcarnitine and 3-hydroxy acylcarnitine molecular species in diabetic myocardium: Identification of alterations in mitochondrial fatty acid processing in diabetic myocardium by shotgun lipidomics. Biochemistry 44:5234–5245. https://doi.org/10.1021/bi047773a
10. Tonin AM, Amaral AU, Busanello ENB, et al (2013) Long-chain 3-hydroxy fatty acids accumulating in long-chain 3-hydroxyacyl-CoA dehydrogenase and mitochondrial trifunctional protein deficiencies uncouple oxidative phosphorylation in heart mitochondria. J Bioenerg Biomembr 45:47–57. https://doi.org/10.1007/s10863-012-9481-9
11. Newgard CB, An J, Bain JR, et al (2009) A Branched-Chain Amino Acid-Related Metabolic Signature that Differentiates Obese and Lean Humans and Contributes to Insulin Resistance. Cell Metab. https://doi.org/10.1016/j.cmet.2009.02.002
12. Huffman KM, Shah SH, Stevens RD, et al (2009) Relationships between circulating metabolic intermediates and insulin action in overweight to obese, inactive men and women. Diabetes Care. https://doi.org/10.2337/dc08-2075
13. Wang TJ, Larson MG, Vasan RS, et al (2011) Metabolite profiles and the risk of developing diabetes. Nat Med. https://doi.org/10.1038/nm.2307
14. Pedersen HK, Gudmundsdottir V, Nielsen HB, et al (2016) Human gut microbes impact host serum metabolome and insulin sensitivity. Nature 535:376–381. https://doi.org/10.1038/nature18646
15. Kappel BA, Federici M (2019) Gut microbiome and cardiometabolic risk. Rev Endocr Metab Disord 20:399–406. https://doi.org/10.1007/s11154-019-09533-9
16. Singh H, Miyamoto S, Darshi M, et al (2020) Gut microbial changes in diabetic db/db mice and recovery of microbial diversity upon pirfenidone treatment. Microorganisms 8:1–17. https://doi.org/10.3390/microorganisms8091347
17. Bhattacharya S, Granger CB, Craig D, et al (2014) Validation of the association between a branched chain amino acid metabolite profile and extremes of coronary artery disease in patients referred for cardiac catheterization. Atherosclerosis 232:191–196. https://doi.org/10.1016/j.atherosclerosis.2013.10.036
18. Shah SH, Sun JL, Stevens RD, et al (2012) Baseline metabolomic profiles predict cardiovascular events in patients at risk for coronary artery disease. Am Heart J. https://doi.org/10.1016/j.ahj.2012.02.005
19. Neinast MD, Jang C, Hui S, et al (2019) Quantitative Analysis of the Whole-Body Metabolic Fate of Branched-Chain Amino Acids. Cell Metab 29:417-429.e4. https://doi.org/10.1016/j.cmet.2018.10.013
20. Sun H, Olson KC, Gao C, et al (2016) Catabolic defect of branched-chain amino acids promotes heart failure. Circulation 133:2038–2049. https://doi.org/10.1161/CIRCULATIONAHA.115.020226
21. Kappel BA, Lehrke M, Schütt K, et al (2017) Effect of Empagliflozin on the Metabolic Signature of Patients With Type 2 Diabetes Mellitus and Cardiovascular Disease. Circulation 136:969–972. https://doi.org/10.1161/CIRCULATIONAHA.117.029166
22. Moellmann J, Klinkhammer BM, Droste P, et al (2020) Empagliflozin improves left ventricular diastolic function of db/db mice. Biochim Biophys Acta - Mol Basis Dis 1866:. https://doi.org/10.1016/j.bbadis.2020.165807
23. Santos-Gallego CG, Requena-Ibanez JA, San Antonio R, et al (2019) Empagliflozin Ameliorates Adverse Left Ventricular Remodeling in Nondiabetic Heart Failure by Enhancing Myocardial Energetics. J Am Coll Cardiol 73:1931–1944. https://doi.org/10.1016/j.jacc.2019.01.056
