# Supplementary material for: Human and mouse non‐targeted metabolomics identify 1,5‐anhydroglucitol as SGLT2‐dependent glycemic marker
Source: Clin Transl Med. 2021 Jun 27;11(6):e470. doi: 10.1002/ctm2.470 (PMC8236115; doi:10.1002/ctm2.470)
Supplement: Supplementary file 4 — Supporting Information [file CTM2-11-e470-s004.pdf]

SUPPLEMENTARY METHODS

Experimental animals

Animal experiments were approved by the government of North Rhine-Westphalia, Germany. The mouse study was conducted as previously described [1]. Male Db/Db mice (BKS(D)-Leprdb/db/JOrlRj) and Db/+ control mice (BKS(D)-Leprdb/+/JOrlRj) were purchased from Janvier labs at the age of 6 weeks. Mice were housed in shoebox-sized filtertop cages in a controlled environment (12h daylight cycle) at the animal facility of the RWTH Aachen University. Mice had free access to water and food. Mice were fed a high-fat Western-type diet (39 kJ% fat, 41 kJ% carbohydrates and 20 kJ% protein; ssniff EF R/M acc. TD88137AQ8 mod.; ssniff Spezialdiäten GmbH) for 4.5 weeks. A group of Db/Db mice further received empagliflozin in a concentration of 150 mg/kg. Overnight fasted mice were sacrificed at the end of the experiment. Serum was snap-frozen and stored at -80°C until metabolomics analysis.

Phenotyping of these mice has been described in detail in a previous publication [1]. Db/Db mice compared to Db/+ revealed higher body weight, increased blood glucose as well as an altered lipid profile. Empagliflozin reduced serum glucose by increasing urinary glucose excretion. Cardiac functional analysis revealed diastolic dysfunction in Db/Db mice, which was improved by empagliflozin treatment [1].

Myocardial infarction (MI) cohort

Patients were recruited in a prospective cohort study (all subjects gave written informed consent, validated and approved by the ethical committee of Policlinico Tor Vergata University of Rome, “AMIDIAB study 28/4/2008”) at the Policlinico Tor Vergata between 02/2015 and 04/2016. Inclusion criteria were hospitalization for type 1 MI according to the Third Universal Definition of Myocardial Infarction (ST-elevation myocardial infarction (STEMI) or non-ST segment elevation myocardial infarction (NSTEMI)) with indication for coronary angiography. 40 patients were enrolled in the study. 3 patients were excluded from the metabolomics analysis due to the lack of information on the diagnosis of type 2 diabetes. Clinical data were recorded and serum was taken during the hospital stay at 7 a.m. Sample size was not determined by statistical methods, given the exploratory nature of the project. Baseline data are provided in Supplementary Table S1. STROBE diagram is shown in Supplementary Figure S1.

Empagliflozin registry

Patients were recruited in a prospective cohort study (Trial registration: NCT03131232. Registered 24 April 2017 - Retrospectively registered, https://clinicaltrials.gov/ct2/show/NCT03131232; approved by the ethics committee of the Medical Faculty of RWTH Aachen University, and all patients gave informed consent) at the RWTH Aachen University Hospital between 12/2015 and 07/2016 as previously described [2]. Inclusion criteria were cardiovascular disease and established diagnosis of type 2 diabetes with indication for intensification of glucose-lowering therapy. Patients with advanced chronic kidney disease (estimated glomerular filtration rate (eGFR) below 45ml/min), active infection, acute cardiovascular event and type 1 diabetes mellitus were excluded from the study. All patients received empagliflozin 10 mg once daily. Clinical data were recorded and serum was taken at baseline before treatment as well as at a one month and six months visit. 42 patients were enrolled in the study. 17 patients were excluded from the metabolomics analysis for one of the following reasons: no serum available at baseline and/or one month follow up, lost before one month follow up, discontinuation of study drug or withdrawal of consent. 25 patients were included in the metabolomics analysis: 14 patients with serum available at baseline, 1 month follow up and 6 months follow up and 11 patients with serum available only at baseline and 1 month follow up. Sample size was not determined by statistical methods, given the exploratory nature of the project. Baseline data are provided in Supplementary Table S2. STROBE diagram is shown in Supplementary Figure S2.

Non-targeted metabolomics (MI cohort and Empagliflozin registry)

Metabolomics analysis of mouse serum samples and of the empagliflozin registry were conducted as described previously [1, 2]. Samples were stored at -80 °C prior to analysis at Helmholtz Zentrum München, Germany. On the day of extraction, samples were thawed on ice and randomized.

A hundred µL of the serum were pipetted into a 2 mL 96-well plate. In addition to samples of the study, a 100 µL human reference EDTA plasma sample (Seralab, West Sussex, UK) and 100 µL human reference serum samples (Seralab, West Sussex, UK) were placed in 1 and 6 wells, respectively, of each of the 96-well plate. These samples served as technical replicates throughout the data set to

assess process variability. Water samples, serve as process blank, were also placed into 6 wells (100 µL each) of each plate.

Protein was precipitated and the metabolites in the samples were extracted with 475 µL methanol, containing four recovery standard compounds to monitor the extraction efficiency. After centrifugation, the supernatant of each sample was split into 4 aliquots of 100 µL each onto two 96-well microplates. Metabolomics analysis in mice and human MI cohort: the first 2 aliquots were used for reverse phase (RP)/UPLC-MS/MS analysis in positive and negative electrospray ionization (ESI) mode. Two further aliquots on the second plate were kept as a reserve. The samples were dried on a TurboVap 96 (Zymark, Sotax, Lörrach, Germany). Prior to the UPLC-MS/MS in positive ion mode, the samples were reconstituted with 50 µL of 0.1% formic acid (FA) and those analyzed in negative ion mode with 50 µL of 6.5 mM ammonium bicarbonate, pH 8.0. Reconstitution solvents for both ionization modes contained further internal standards that allowed monitoring of instrument performance and also served as retention reference markers. Metabolomics analysis in the empagliflozin registry: 2 aliquots were used for analysis by 2 separate RP/UPLC-MS/MS methods with positive ion mode ESI, 1 for analysis by RP/UPLC-MS/MS with negative ion mode ESI, and 1 for analysis by (HILIC)/UPLC-MS/MS with negative ion mode ESI.

To minimize human error, liquid handling was performed on an automated MicroLab STAR® robot (Hamilton Bonaduz AG, Bonaduz, Switzerland). All UPLC-MS/MS analysis were performed on a Q Exactive high resolution/accurate mass spectrometer (Thermo Fisher Scientific GmbH, Dreieich, Germany) coupled with a Waters Acquity UPLC system (Waters GmbH, Eschborn, Germany).

Metabolomics analysis in mice and human MI cohort: two separate columns (2.1 x 100 mm Waters BEH C18 1.7 µm particle) were used for acidic (solvent A: 0.1% FA in water, solvent B: 0.1% FA in methanol) and for basic (A: 6.5 mM ammonium bicarbonate pH 8.0, B: 6.5 mM ammonium bicarbonate in 95% methanol) mobile phase conditions, optimized for positive and negative ESI, respectively. After injection of the sample extracts, the columns were developed in a gradient of 99.5% A to 98% B in 11 min run time at 350 µL/min flow rate. The eluent flow was directly connected to the ESI source of the mass spectrometer. Full scan mass spectra (80 – 1000 m/z) and data dependent MS/MS scans with dynamic exclusion were recorded in turns.

Metabolomics analysis in the empagliflozin registry: prior to the UPLC-MS/MS runs the dried extract samples were reconstituted with 80 µL of solvents compatible to each of the 4 methods. Each

reconstitution solvent contained a series of standards at fixed concentrations to ensure injection and chromatographic consistency. One aliquot was analyzed using acidic positive ion conditions, chromatographically optimized for more hydrophilic compounds. In this method, the extract was gradient eluted from a C18 column (Waters UPLC BEH C18-2.1 x 100 mm, 1.7 µm) using water and methanol, containing 0.05% perfluoropentanoic acid (PFPA) and 0.1% FA. Another aliquot was also analyzed using acidic positive ion conditions; however it was chromatographically optimized for more hydrophobic compounds. In this method, the extract was gradient eluted from the same afore mentioned C18 column using methanol, acetonitrile, water, 0.05% PFPA and 0.01% FA and was operated at an overall higher organic content. Another aliquot was analyzed using basic negative ion optimized conditions using a separate dedicated C18 column. The basic extracts were gradient eluted from the column using methanol and water, however with 6.5 mM ammonium bicarbonate at pH 8. The fourth aliquot was analyzed via negative ionization following elution from a HILIC column (Waters UPLC BEH Amide 2.1x150 mm, 1.7 µm) using a gradient consisting of water and acetonitrile with 10 mM ammonium formate, pH 10.8. The MS analysis alternated between MS and data-dependent MSn scans using dynamic exclusion. The scan range varied slighted between methods but covered 70-1000 m/z.

For all metabolomics studies, raw data was extracted, peak-identified and QC processed using Metabolon's hardware and software (Metabolon, Inc., North Carolina, USA). Metabolites were annotated by curation of the LC-MS/MS data against proprietary Metabolon's chemical database library (Metabolon, Inc., Durham, NC, USA) based on retention index, precursor mass and MS/MS spectra.

Placebo-controlled, randomized, double blind human trial with empagliflozin

Patients were recruited in a prospective randomized placebo-controlled double blind study at the RWTH Aachen University Hospital between 05/2017 and 10/2018 (Trial registration: EudraCT Number: 2016-000172-19; registered 21 November 2016; https://www.clinicaltrialsregister.eu/ctr-search/trial/2016-000172-19/DE; approved by the ethics committee of the Medical Faculty of RWTH Aachen University and the German Federal Institute for Drugs and Medical Devices (Bundesinstitut für Arzneimittel und Medizinprodukte, BfArM), all patients gave informed consent). Study design, inclusion/exclusion criteria, randomization, baseline characteristics and outcome of this trial have been described previously [3]. In short, patients with type 2 diabetes were randomized to placebo (n = 22) or empagliflozin 10mg once

daily (n = 20). Targeted analysis of 1,5-anhydroglucitol in patients' serum was performed at baseline, 72 hours and 3 months after trial initiation.

Targeted measurement of 1,5-anhydroglucitol (placebo-controlled human trial)

Targeted measurement of 1,5-anhydroglucitol has been performed at the Katholieke Universiteit Leuven Metabolomics Expertise Center. Serum metabolites were extracted by adding 10 uL of serum to 990 uL of an 80% methanol (in water) extraction buffer containing 2 uM of deuterated (d27) myristic acid as internal standard). Following extraction overnight at -80 degrees Celsius. Precipitated proteins and insolubilities were removed by centrifugation at 20.000 x g for 20 min at 4 degrees Celsius. The supernatant was transferred to the appropriate mass spectrometer vials. Measurement was performed using a Vanquish LC System (Thermo Scientific) in-line connected to a Lumos Orbitrap mass spectrometer (Thermo Scientific). 10 ul of sample was injected and concentrated on an InfinityLab Poroshell 120 HILIC-Z, 2.1 x 150 mm, 2.7 µm, PEEK-lined column (Agilent). A linear gradient was carried out starting with 90% solvent A (LC-MS grade acetonitrile) and 10% solvent B (10 mM ammoniumacetate pH 9.3). From 2 to 12 minutes the gradient changed to 60% B and was kept at 60% until 15 min. Next a decrease to 10% B was carried out to 16 min and remained at 10% B until 25 min. The solvent was used at a flow rate of 250 ul/min, the columns temperature was kept constant at 25 degrees Celsius. The mass spectrometer operated in negative ion mode, settings of the HESI probe were as follows: sheath gas flow rate at 40, auxiliary gas flow rate at 10. Spray voltage was set at 3.2 kV, temperature of the capillary at 300 degrees Celsius and S-lens RF level at 50. A full scan (resolution of 240.000 and scan range of m/z 70-750) was applied. For data analysis a Compound Discoverer 3.0 (Thermo Scientific) software platform for analyzing metabolites was used. 1,5-anhydroglucitol was identified according to a in-house library.

Statistical analysis

Data were analyzed and visualized using GraphPad Prism 8.0, IBM SPSS Statistics Version 26 and MetaboAnalyst 4.0 [4]. Statistical analyses were carried out using unpaired two-tailed Student's t-test, one-way analysis of variance (ANOVA) with post hoc tests and Chi square test, when appropriate. Data are presented as means ± standard deviation (S.D.). A P-value < 0.05 was considered to be statistically

significant. Metabolomics data were corrected for multiple testing by false discovery rate (FDR) approach.

REFERENCES

1. Moellmann J, Klinkhammer BM, Droste P, et al (2020) Empagliflozin improves left ventricular diastolic function of db/db mice. Biochim Biophys Acta - Mol Basis Dis 1866:. https://doi.org/10.1016/j.bbadis.2020.165807
2. Kappel BA, Lehrke M, Schütt K, et al (2017) Effect of Empagliflozin on the Metabolic Signature of Patients With Type 2 Diabetes Mellitus and Cardiovascular Disease. Circulation 136:969–972. https://doi.org/10.1161/CIRCULATIONAHA.117.029166
3. Rau M, Thiele K, Hartmann NUK, et al (2021) Empagliflozin does not change cardiac index nor systemic vascular resistance but rapidly improves left ventricular filling pressure in patients with type 2 diabetes: a randomized controlled study. Cardiovasc Diabetol 20:1–12. https://doi.org/10.1186/s12933-020-01175-5
4. Xia J, Wishart DS (2016) Using MetaboAnalyst 3.0 for Comprehensive Metabolomics Data Analysis. Curr Protoc Bioinforma 14.10.1-14.10.91. https://doi.org/10.1002/cpbi.11
